# Obstructive Sleep Apnea as a Risk Factor for Atrial Fibrillation: A Meta-Analysis

**DOI:** 10.4172/2167-0277.1000282

**Published:** 2018-02-12

**Authors:** Irini Youssef, Haroon Kamran, Mena Yacoub, Nirav Patel, Clive Goulbourne, Shweta Kumar, Jesse Kane, Haley Hoffner, Moro Salifu, Samy I McFarlane

**Affiliations:** 1 Department of Medicine, Divisions of Endocrinology, Cardiology and Renal Medicine, State University of New York, Downstate Medical Center, Brooklyn, NY, USA; 2 Department of Cardiology, Northside Hospital, St. Petersburg FL, USA; 3 Department of Cardiology, Hartford Hospital, Hartford, CT

**Keywords:** Sleep Apnea, Atrial Fibrillation, Meta-analysis, Health, Cardiac arrhythmias

## Abstract

**Objectives:**

To conducted a meta-analysis assessing the relationship between Obstructive Sleep Apnea (OSA) and the risk of Atrial Fibrillation (AF)

**Methods:**

We searched PUBMED, Medline, and Cochrane Library using the keywords “atrial fibrillation”, “obstructive sleep apnea” and “sleep disordered breathing (SDB)”. All subjects included had established diagnosis of OSA/SDB. We then compared the occurrence of AF versus no AF. Analysis done with Comprehensive Meta-Analysis package V3 (Biostat, USA).

**Results:**

A total of 579 results were generated. Duplicates were removed and 372 records were excluded based on irrelevant abstracts, titles, study design not consistent with the stated outcome, or full-text unavailable. Twelve studies meeting the inclusion criteria were reviewed in full-text; 2 of these articles were eventually removed due to unconfirmed OSA diagnostic modality, and one was also removed based on a control group inconsistent with the other studies. Therefore, a total of 9 studies were included (n=19,837). Sample sizes ranged from n=160 patients to n=6841 patients. The risk of AF was found to be higher among OSA/SDB versus control group (OR; 2.120, C.I: 1.845–2.436, Z; 10.598 p: <0.001). The heterogeneity observed for the pooled analysis was Q-value; 22.487 df (Q); 8 P-value; 0.004, I-squared; 64.424 Tau2; 0.098, suggesting appropriate study selection and moderate heterogeneity.

**Conclusion:**

OSA/SDB is strongly associated with AFib confirming the notion that OSA/SDB populations are high risk for development of AF. Prospective studies are needed to ascertain the effect of the treatment of OSA/SDB for the prevention of AF, a growing health burden with serious consequences.

## Introduction

AF is a growing health problem worldwide, estimated to affect 5.6 million to 15.9 million people in the United States by 2050 [1]. It is associated with an increased risk for morbidity and mortality [2], Modifiable risk factors for AF have been delineated, and include hypertension, obesity and alcohol consumption [3]. As the incidence of AF continues to rise, it is imperative to identify and treat potentially modifiable risk factors for the disease. OSA/SDB has been identified as potentially modifiable risk factors for AF. OSA is generally under-diagnosed medical problem, estimated to affect as many as 1 in 5 adults with the incidence on the rise as the obesity epidemic continues to grow [4]. OSA is associated with a myriad of negative health consequences including hypertension, heart disease, depression, and premature mortality [5]. Growing evidence suggests that OSA may be implicated in providing both a substrate and maintenance factor for AF [6]. There have been several proposed hypothesis as well as animal models attempting to clarify the role of the underlying pathophysiological mechanisms of OSA on the genesis of AF; however, there are little or no controlled studies of all relevant available data, showing a direct impact of OSA on the incidence of AF.

We now aim to extend prior work explaining the relationship between OSA and AF by performing a comprehensive meta-analysis reporting the association between OSA and AF.

The objective of this meta-analysis is to integrate all available data in an effort to identify a role, if any, of OSA on the development or progression of AF.

## Methods

We searched databases including PUBMED, Medline, and Cochrane Library for relevant studies using the keywords “atrial fibrillation” and “obstructive sleep apnea”. Relevant references cited in other studies were also scrutinized for the study objective. Studies included in our meta-analysis provided odds-ratio or relative-risk values with 95% confidence intervals (CIs).

The OSA group, included an apnea-hypopnea index of ≥ 5 as diagnostic of OSA or 3% oxygen desaturation index (ODI) of >15 events, or a respiratory disturbance index (RDI) of >30 events. The Control group for all studies was defined as no recent or prior diagnosis of OSA by history or diagnostic testing (all indices <5 events).

**Figure 1:**
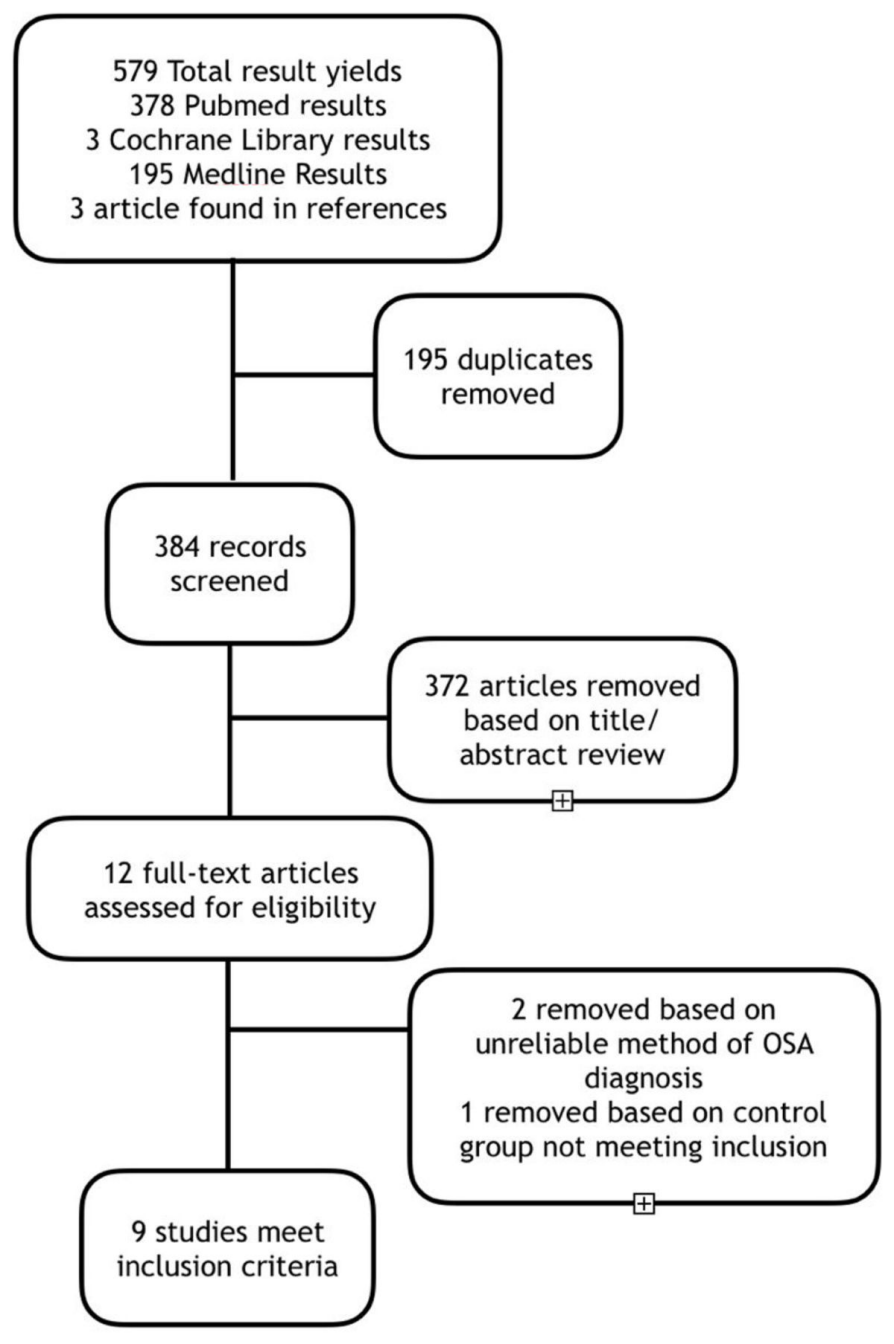
Flow diagram explaining the process of selections of the studies included in our meta-analysis.

To qualify for inclusion, the study must have (1) designated OSA as the exposure; (2) have a proper control group (no OSA) as mentioned; (3) have atrial fibrillation as the primary outcome, or an outcome amongst other diagnosed arrhythmia; (4) must have mentioned its criteria for diagnosing OSA, and the method of diagnosis. Studies excluded were studies not answering the objective. Additional exclusion criteria included (1) studies identifying atrial fibrillation as the exposure; (2) studies failing to clarify their definition of obstructive sleep apnea; (3) studies that did not specify atrial fibrillation in their arrhythmias of interest; (4) studies including patients treated with CPAP; (5) absence of a “non-exposed” (control) group; and (6), data only reported in abstract form Figure 1.

The following data was collected from each study (1) OSA criteria; (2) total study population; (3) number of subjects in “exposed” and “non-exposed” group; (4) number of subjects who developed AF per group;(4) P values and CI’s.

### Data analysis

The search of the databases and references yielded a total of 579 results. After removing duplicate studies, there were 384 studies left for screening and 372 of these records were excluded (Figure 1), based on not meeting the inclusion criteria. Twelve studies were read by full-text; 2 of these articles were removed due to unmentioned or unreliable OSA diagnostic modality, and 1 was removed based on a control group inconsistent with other studies (e.g. AHI >15 diagnostic for OSA). This left us with 9 studies of 19,837 patients that met all inclusion criteria, 12,255 exposed, and 7582 non-exposed. Sample sizes ranged from n=160 patients to n=6841 patients. Characteristics of these studies were as follows: 4 cross sectional studies, 3 prospective cohort studies, and 2 retrospective cohort studies.

For the purpose of this analysis the OSA group included any severity of OSA; namely mild, moderate or severe OSA. This was important to minimize the variability of data among individual studies, as some studies included mild vs. no OSA while others reported moderate to severe vs. no OSA.

We used Comprehensive Meta-Analysis package V3 (Biostat, USA). Mantel-Haenszel method (Mantel & Haenszel) for calculating the weighted pooled odds ratio under the fixed effects model. We incorporated heterogeneity statistics to calculate the summary odds ratio under the random effects model (Der Simonian & Laird). The data is reported as odds ratios with 95% Confidence intervals using the random effects model.

## Results

**Figure 2:**
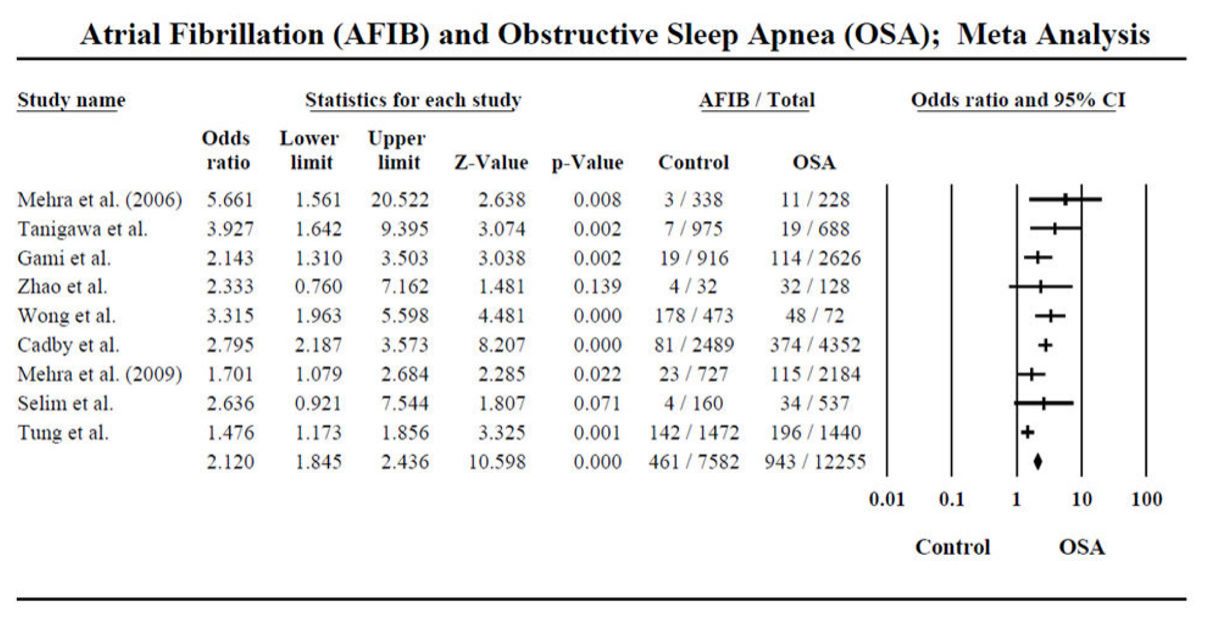
Forrest plot depicting the odds ratio of having AFIB associated with OSA.

There were a total of 9 studies included in this meta-analysis for the random pooled effects. In the main pooled analysis, the control group included 7582 subjects, of which 461 developed AF and the OSA group included 12255 subjects out of which 943 developed AF. The risk of AF was found to be higher among OSA versus control group (OR; 2.120, C.I: 1.845–2.436, Z; 10.598 p<0.001). These results suggest higher incidence of AF among patients with an established diagnosis of OSA vs. no OSA (Figure 2).

2 out of the 9 studies showed no difference between OSA and no OSA and incidence of AF (Zhao et al. (OR: 2.333, C.I: 0.760–7.162, Z: 1.481 p<0.139) and Selim et al. (OR; 2.636, C.I: 0.921–7.544, Z; 1.807 p<0.071).

The Heterogeneity observed for the pooled analysis was Q-value; 22.487 df (Q); 8 P-value; 0.004, I-squared; 64.424 Tau2; 0.098, suggesting appropriate study selection and moderate heterogeneity (Table 1).

## Discussion

The results of our study demonstrate that OSA/SDB is strongly associated with atrial fibrillation. Our results are consistent with other large-scale studies that have found an independent and strong association between SDB and cardiac arrhythmias. Cadby et al. studied patients attending a sleep clinic referred for in-laboratory polysomnography for possible OSA between 1989 and 2001. After adjustment for multivariate predictors, the authors found that OSA diagnosis (AHI>5), OSA severity (using AHI clinical cut points), log (AHI+1), and log (SaO2t<90%+1) were all independently predictive of developing AF. Their adjusted HR for AHI>5 vs. AHI<5 was 1.55 (95% CI, 1.21–2.00).

Gami et al. is another large-scale retrospective cohort study, which determined the incidence of AF in patients referred for polysomnography. In their study of 3,542 patients, AF occurred in 114 of 2,626 patients (4.3%) with OSA and in 19 of 916 patients (2.1%) without OSA. Moreover, OSA (defined by an apnea-hypopnea index 5) was a strong predictor of incident AF (HR 2.18, 95% CI 1.34 to 3.54, p 0.002). Additionally, the authors also found that the severity of OSA was associated with higher incidence of incident AF.

Of the nine studies included in this analysis, two did not reach statistical significance. The Sleep apnea and Atrial fibrillation after Bypass OperaTion (SABOT) study was a prospective observational study conducted on 160 patients without AF who underwent elective CABG. The authors of the study measured the prevalence of OSA in patients prior to CABG then followed the patients post-operatively for the occurrence of incident AF.

There have been several postulated mechanisms for the role of obstructive sleep apnea as a promulgator of atrial fibrillation. Hypoxia induced chemo-reflexive tachycardia and hypertension may increase myocardial oxygen demand resulting in repeated atrial ischemia and subsequent AF [16]. Animal models have shown that hypoxia induces transient prolongation and an increase in heterogeneity of refractory periods, depressed conduction velocity, and increased heterogeneous conduction, causing expanded vulnerability to re-entrant arrhythmias [17]. Increased sympathetic tone and decreased parasympathetic tone have been found to precede generation of paroxysmal AF. These autonomic changes are secondary to recurrent nocturnal apneas and chemoreceptor induced decrease in parasympathetic tone, manifesting as impaired cardiac vagal input and parasympathetic components of heart rate variability [18].

Upper airway collapse during sleep also generates major drops in intrathoracic pressure that are transmitted to the thin-walled atria, contributing to atrial chamber enlargement secondary to increased transmural forces. These changes are associated with greater left atrial volumes and a greater degree of left ventricular diastolic dysfunction in controls without OSA, even after matching for BMI [19]. These same forces may be important in tissue stretching at the level of pulmonary vein ostia, foci commonly implicated in development of AF [20].

Repeated cycles of hypoxia and deoxygenation also lead to the formation of reactive oxygen species, and oxidative stress has been implicated as a substrate of AF initiation and maintenance [21]. Markers of inflammation, including CRP, IL-6 and IL-8, are also increased in chronic OSA, and have been associated with a greater risk of AF, post-CABG AF, and AF recurrence after cardio version and ablation [22].

Finally, elevated levels of Angiotensin II and Aldosterone, found in human and animal studies during increased sympathetic activation, and completely mitigated by renal sympathetic denervation, in combination with reactive oxygen species, potentially contribute to atrial tissue fibrosis and arrhythmogenesis [23].

Despite the rapidly growing epidemic of obesity and attendant risk of OSA, the US Preventive Services Task Force conclude that the current evidence is insufficient to assess the balance of benefits, and harms of screening for OSA in asymptomatic adults. Given the increasing prevalence of OSA risk factors including obesity, the current prevalence of OSA may be underestimated. Thus, further research on subclinical OSA and its risk factors (male sex, older age, postmenopausal status, higher BMI, cranio-facial and upper airway abnormalities) is required in order to select patients most likely to benefit from screening, and early treatment, to prevent or decrease likelihood of AF development or progression [24].

## Limitations

There are some limitations to our meta-analysis. A few of our studies were cross-sectional data, limiting the ability to draw conclusions about temporality and incident risk. One of the studies in our meta-analysis determined exposure status based on review of subject medical records. The authors did not specifically mention the diagnostic test used to determine the presence of OSA in these patients. Nevertheless, removal of this study during data-analysis did not alter the results of our study, and so it was still included. Mehra et al. sampled the extremes of the SDB spectrum (SDB defined as RDI>30 events/h and non-SDB defined as RDI<5), which increased the efficiency of the study, but limited the ability to evaluate an intermediate threshold (between RDI of 5 and 30). Moreover, some studies consisted of samples of patients referred to sleep clinics because of suspected sleep disorders. This may lead to a potential ascertainment bias. Another limitation is that patients in prospective cohort studies have greater follow-up compared to patients with OSA in the general population, which could have led to increased diagnosis of AF in study patients compared to symptomatic AF patients. Finally, the Mehra et al. study included a population of purely males, precluding the opportunity of extending the results to female populations.

The utility of the apnea-hypopnea index as an accurate assessment of OSA has its inherent limitations; the mean time of each apneic and hypopneic episode is not included in the index. Moreover, due to the combination of both episodes into one index, the comparability of patients with more apneic versus hyopneic episodes may lead to an underestimation of OSA severity in those with more or longer apneic episodes due to longer and greater reductions in airflow and arterial oxygen saturation [15].

In all, this study brings to light an important risk factor for one of the most common and globally burdening cardiac arrhythmia. An increased awareness of this association is tantamount to identification and treatment of OSA to decrease its impact on the development of AF.

## Figures and Tables

**Table 1:** Study characteristics.

Study	Type of Study	Number of patients	Number of exposed	Number of non-exposed	Total Afib	AF in non-exposed	AF in exposed	P value/CI
Mehra et al. [7]	Cross-sectional	566	228	338	14	3	11	1.03–15.74
Tanigawa et al. [8]	Prevalence	1663	688	975	26	7	19	P<0.001
Gami et al. [9]	Retrospective cohort	3542	2626	916	133	19	114	1.34 to 3.54
Zhao et al. [10]	prospective	160	128	32	36	4	32	p=0.07
Wong et al. [11]	Retrospective	545	72	473	226	178	48	1.3–2.58
Cadby et al. [12]	Prospective	6841	4352	2489	455	81	374	2.06–3.34
Mehra et al. [13]	cross-sectional	2911	2184	727	138	23	115	P<0.001
Selim et al. [14]	Cross-sectional	697	537	160	38	4	34	P>0.05
Tung et al. [15]	Prospective	2912	1440	1472	338	142	196	P>0.05
